# Climate of the Western Hemisphere

**Published:** 1915-10

**Authors:** 


**Climate of the Western Hemisphere.** Temperature (Degrees Fahrenheit). Prudential Life Ins. Co

| Country | City | Jan. to March | April to June | July to Sept. | Oct. to Dec. | Annuel |
|---|---|---|---|---|---|---|
| British Guiana | Georgetown | 79.3 | 80.3 | 80.8 | 80.7 | 80.3 |
| Philippine Islands | Manila | 78.2 | 82.8 | 80.7 | 78.8 | 80.1 |
| Barbados | Bridgetown | 78.7 | 80.4 | 80.9 | 79.9 | 80.0 |
| Dutch Guiana | Paramaribo | 78.5 | 79.1 | 80.3 | 80.2 | 79.5 |
| Panama | Colon | 79.1 | 80.0 | 79.4 | 78.8 | 79.4 |
| Brazil | Para | 78.5 | 78.9 | 79.5 | 80.3 | 79.3 |
| British Honduras | Belize | 76.9 | 81.4 | 82.4 | 76.4 | 79.3 |
| Nicaragua | San Juan del Norte | 78.0 | 80.6 | 79.6 | 78.7 | 79.2 |
| Brazil | Manaos | 78.1 | 78.4 | 79.2 | 80.1 | 79.0 |
| Jamaica | Kingston | 76.0 | 79.7 | 81.1 | 78.3 | 78.8 |
| Grenada | Richmond Hill | 78.4 | 78.5 | 79.3 | 79.0 | 78.8 |
| Trinidad | Port of Spain | 78.2 | 79.7 | 77.3 | 78.5 | 78.4 |
| Brazil | Bahia | 80.7 | 77.9 | 75.1 | 79.0 | 78.2 |
| Porto Rico | San Juan | 75.4 | 78.5 | 80.5 | 78.3 | 78.2 |
| Saint Vincent | Kingstown | 76.5 | 78.1 | 78.7 | 78.6 | 78.0 |
| Cuba | Habana | 71.8 | 78.8 | 81.8 | 74.9 | 76.8 |
| Paraguay | Asuncion | 84.4 | 68.9 | 68.8 | 79.2 | 75.3 |
| Hawaii | Honolulu | 71.0 | 75.0 | 78.1 | 74.3 | 74.6 |
| Brazil | Rio de Janeiro | 76.9 | 71.6 | 69.7 | 74.3 | 73.1 |
| Venezuela | Caracas | 68.9 | 73.1 | 72.4 | 70.5 | 71.2 |
| U. S. | New Orleans | 61.3 | 75.2 | 81.5 | 63.0 | 70.3 |
| Bermuda | Hamilton | 62.5 | 69.6 | 78.9 | 68.4 | 69.9 |
| Brazil | Bello Horizonte | 72.4 | 65.8 | 64.1 | 70.6 | 68.2 |
| Costa Rica | San Juan | 66.8 | 68.6 | 67.6 | 66.7 | 67.4 |
| Peru | Lima | 72.5 | 66.0 | 60.8 | 65.8 | 66.2 |
| Argentina | Tucuman | 74.4 | 58.5 | 57.2 | 72.3 | 65.6 |
| Chile | Arica | 71.8 | 65.2 | 59.9 | 65.3 | 65.5 |
| U. S. | Los Angeles | 56.2 | 62.1 | 69.6 | 61.3 | 62.3 |
| Argentina | Buenos Aires | 72.6 | 56.4 | 53.1 | 67.0 | 62.2 |
| Brazil | Curityba | 67.9 | 57.6 | 56.2 | 63.6 | 61.4 |
| Uruguay | Montevideo | 70.2 | 56.8 | 52.0 | 64.1 | 60.8 |
| Mexico | Mexico City | 56.8 | 64.4 | 61.8 | 56.6 | 59.9 |
| Argentina | Bahia Glanca | 69.3 | 53.6 | 49.6 | 62.7 | 58.8 |
| Colombia | Bogota | 58.0 | 58.4 | 57.1 | 58.1 | 57.9 |
| Chile | Santiago | 66.4 | 51.4 | 48.3 | 61.4 | 56.9 |
| U. S. | San Francisco | 51.4 | 56.0 | 58.2 | 55.6 | 55.3 |
| U. S. | Washington | 39.0 | 62.8 | 73.2 | 45.7 | 55.2 |
| Ecuador | Quito | 54.7 | 54.7 | 54.9 | 54.6 | 54.7 |
| U. S. | Denver | 37.6 | 56.3 | 68.8 | 40.5 | 50.8 |
| Peru | Cuzco | 51.5 | 49.8 | 48.9 | 52.1 | 50.6 |
| U. S. | Chicago | 32.6 | 57.2 | 71.2 | 41.4 | 50.6 |
| U. S. | Boston | 32.0 | 57.2 | 68.9 | 43.4 | 50.4 |
| Bolivia | La Paz | 49.0 | 45.4 | 48.8 | 51.4 | 48.7 |
| Canada | Vancouver | 37.9 | 52.7 | 60.2 | 42.9 | 48.5 |
| Falkland Islands | Port Stanley | 53.5 | 43.1 | 39.1 | 47.0 | 45.7 |
| Canada | Toronto | 24.5 | 53.0 | 66.8 | 38.2 | 45.6 |
| Chile | Punta Arenas | 52.9 | 40.8 | 37.8 | 48.7 | 45.1 |
| Canada | Halifax | 25.1 | 48.2 | 62.5 | 38.9 | 43.7 |
| Canada | Montreal | 16.6 | 53.2 | 65.0 | 33.6 | 42.1 |
| Canada | St. John | 22.7 | 47.5 | 59.8 | 37.3 | 41.8 |
| Newfoundland | St. Johns | 24.7 | 41.9 | 57.0 | 37.5 | 40.3 |
| Canada | Edmonton | 14.4 | 48.9 | 58.1 | 27.5 | 37.2 |
| Greenland | Godthaab | 14.9 | 32.7 | 41.4 | 23.7 | 28.2 |
| Alaska | Nome | 4.3 | 32.4 | 47 2 | 16.8 | 25.2 |
| Canada | Dawson | —11.5 | 45.2 | 51.5 | 4.4 | 22.4 |

